# A qualitative exploration of harm reduction in practice by street-based peer outreach workers

**DOI:** 10.1186/s12954-024-01076-w

**Published:** 2024-08-30

**Authors:** Jill Owczarzak, Emily Martin, Noelle Weicker, Imogen Evans, Miles Morris, Susan G. Sherman

**Affiliations:** 1 Johns Hopkins Bloomberg School of Public Health, 624 N. Broadway Ave, Room 739, Baltimore, MD 21205 USA; 2 Johns Hopkins Bloomberg School of Public Health, 624 N. Broadway Ave, Room 163, Baltimore, MD 21205 USA; 3 Tufts Clinical and Translational Science Institute, 35 Kneeland Street, 7th – 11th Floor, Boston, MA USA; 4 Johns Hopkins Bloomberg School of Public Health, 624 N. Broadway Ave, Room 749, Baltimore, MD 21205 USA

**Keywords:** Harm reduction, Peers, Outreach, Substance use

## Abstract

**Background:**

Despite the widespread use of the phrase “harm reduction” and the proliferation of programs based on its principles during the current opioid epidemic, what it means in practice is not universally agreed upon. Harm reduction strategies have expanded from syringe and needle exchange programs that emerged in the mid-1980s primarily in response to the HIV epidemic, to include medication for opioid use disorder, supervised consumption rooms, naloxone distribution, and drug checking technologies such as fentanyl test strips. Harm reduction can often be in tension with abstinence and recovery models to address substance use, and people who use drugs may also hold competing views of what harm reduction means in practice. Street-based outreach workers are increasingly incorporated into harm reduction programs as part of efforts to engage with people more fully in various stages of drug use and nonuse.

**Method:**

This paper explores how peer outreach workers, called “members,” in a street-based naloxone distribution program define and practice harm reduction. We interviewed 15 members of a street-based harm reduction organization in an urban center characterized by an enduring opioid epidemic. Inductive data analysis explored harm reduction as both a set of principles and a set of practices to understand how frontline providers define and enact them.

**Results:**

Analysis revealed that when members talked about their work, they often conceptualized harm reduction as a collection of ways members and others can “save lives” and support people who use drugs. They also framed harm reduction as part of a “path toward recovery.” This path was complicated and nonlinear but pursued a common goal of life without drug use and its residual effects. These findings suggest the need to develop harm reduction programs that incorporate both harm reduction and recovery to best meet the needs of people who use drugs and align with the value systems of implementers.

## Introduction

Harm reduction has become a central pillar of efforts to mitigate the most recent iteration of the opioid overdose epidemic in the United States, an epidemic characterized by an infusion of powerful synthetic opioids into the illicit drug market. The origins of a harm reduction approach can be traced to the Netherlands in the 1970s, when the government began to recognize that strict drug law enforcement itself created social and political harm [42]. In the United States, harm reduction gained increased support over 40 years ago as a public health response to the early HIV epidemic and concern for the health and safety of people who use drugs (PWUD) [12, 15]. In practice, harm reduction as an informal grassroots practice organized by PWUD included distribution of sterile syringes [34] and has since become a pillar of public health policy and practice [5]. Harm reduction strategies have been expanded from syringe and needle exchange programs to include medication for opioid use disorder (MOUD), safe consumption rooms, naloxone distribution, and drug checking such as fentanyl test strips [44, 46].

As a specific strategy, the philosophy and practice of harm reduction in the context of substance use prioritizes reducing the negative consequences of drug use rather than eliminating drug use or requiring or expecting abstinence as a condition of service or a goal [9, 41, 53]. In this way, a harm reduction approach can be seen as in conflict with abstinence and recovery models of addressing substance use. Harm reduction programs promote decreasing the negative effects of drug use for individuals and embrace an ethic of respect for the autonomy of PWUD around the decision to use drugs. In theory, this approach asks harm reduction practitioners to refrain from moralistic judgment. A harm reduction approach may allow for a “continuum of outcomes,” from changing drug injection practices to avoid infections and overdose to complete abstinence. However, abstinence and “recovery” are not explicit goals or policy pillars of a harm reduction approach [23]. Moreover, harm reduction advocates argue that demanding abstinence can further isolate PWUD and contribute to harm, including harms stemming from social stigma around drug use [33]. In contrast, 12-step approaches such as Alcoholics Anonymous, while open to everyone who has a desire to stop consuming alcohol, prioritize abstinence as the goal and sometimes as a condition of treatment [24]. Abstinence or recovery models can also promote acceptance, progress (rather than “perfection”), multiple paths to recovery, and gradual change—principles that may align with a harm reduction approach [24], p. 1153–1154). However, abstinence-based approaches tend to only consider sobriety a success [24], p. 156). In the United States, the broader drug policy and legal frameworks, as well as funding mechanisms and policies and practices of organizations and services further restricts the spaces in which harm reduction is practiced and reinforces a practical and conceptual division between harm reduction and treatment [17, 21]. In some places, harm reduction approaches may even be illegal, creating a context in which abstinence-based approaches prevail, PWUD have limited choices and ability to protect their health, and abstinence and recovery become the idealized goal for both service providers and PWUD [21]. Outcome expectations can be incorporated into PWUD’s narratives of past events and future goals to construct a preferred identity for themselves and others [39, 40, 51].

Despite abundant evidence of its effectiveness at reducing drug-related morbidity and mortality, a harm reduction approach is not universally embraced by policy makers or community members [7, 8, 11, 18, 26, 27, 29–31, 37, 47, 50]. Critics of a harm reduction approach, for example, often argue that it enables and encourages substance use despite the evidence disputing this claim [3]. More recently, overdose prevention sites—locations in which drug use is supervised by medically trained staff and clean injection equipment is provided—have documented success preventing overdose and promoting safer drug use practices [19, 25, 28]. However, expansion of overdose prevention sites has been hindered by discourses of recovery and abstinence, as well as beliefs that such venues would undermine public safety and increase or encourage drug use [4, 8, 43].

In many currently operating harm reduction programs, organizations work toward their goal of reducing harms related to substance use by employing frontline, street-based outreach workers. These workers interact with people on a continuum of drug use, from actively using drugs to no use, and who may or may not use drug treatment programs, access harm reduction services, or express a desire or readiness to quit using drugs. Peers (e.g., peer recovery specialists, peer specialists, peer recovery coaches) extend and enhance the substance use workforce by assisting with health system navigation, case management, and advocacy. Peers can be incorporated into any point on the continuum of drug use, including within harm reduction programs (e.g., syringe service programs, naloxone distribution, drop-in centers) and mutual help groups, and with varying degrees of formality and professional training [45]. Peers share the lived experience of substance use (typically their own, but also that of close family or friends) and can help reduce stigma, motivate the individual, and provide guidance during the recovery process [38]. In addition to helping individuals in recovery navigate the health care system, peers can also provide emotional, instrumental, and informational support [6]. Research also shows that serving in a peer role can benefit the peer through increased self-esteem, personal recovery, employment opportunities, and enhanced “recovery identity” in which their own personal narrative of recovery reinforces their commitment to helping others [49].

This paper explores how peer outreach workers in a street-based naloxone distribution program define and practice harm reduction. It considers harm reduction as both a set of principles and a set of practices, and it explores the ways that frontline providers define and enact them. Understanding how frontline practitioners interpret and enact the fundamental principles of their work has implications for peer and staff recruitment, program implementation, employee retention, and overall program effectiveness.

## Methods

This study took place between April 2018 and April 2019. We interviewed 15 outreach workers of a street-based harm reduction organization that was established in 2017. The outreach workers, called “members,” have recent or current lived experience with drug use – their own or that of people close to them – and are not required to be in recovery as a precondition for hiring. Members conduct outreach and distribute safer drug use supplies (e.g., naloxone, fentanyl test strips, etc.) in neighborhoods experiencing high burden around drug use and overdose. Members receive a small stipend for their work. At the time of data collection, the organization comprised about 30 active staff, including members (outreach workers), supervisors, and administrators, and conducted over 300 outreach events per year. The interviews focused on member work history, experiences with PWUD, feelings about or toward their work, personal histories of and experiences with substance use, ideas about harm reduction, and their recommendations for other activities (if any) that the organization could utilize to further reduce the overdose burden. The study was reviewed and approved by the Institutional Review Board at Johns Hopkins Bloomberg School of Public Health.

All interviews were recorded and transcribed and subsequently uploaded into a qualitative data management and analysis software program (MAXQDA). Working collaboratively, JO and NW developed a coding system for the data. After development, this system was applied to all interviews and captured the main topics from the interviews, as well as unanticipated, emerging topics. Then, this coding system was used to retrieve text excerpts from the interviews as they pertained to the topic of this paper. In addition to identifying any unique experiences or insights, the text segments corresponding to each code were reviewed to identify patterns and common experiences within each domain. Additional details about recruitment, data collection, and analysis have been published elsewhere [35].

## Results

Of the 15 members interviewed, all participants self-identified as black. Four identified as female and 11 identified as male. Participant tenure with the harm reduction organization at the time of the interview ranged from two months to just under four years, with a median tenure of one year.

Members drew on their own lived experience when defining and practicing harm reduction, believing that, once dependent, drugs impact every aspect of a person’s life. They interpreted “harms” holistically to include social and structural harms, in addition to immediate medical harms (e.g., overdose) and detrimental effects to an individual’s personal life and relationships. They framed harm reduction as part of a “path toward recovery” to reconcile the tension between their desire to help people out of drug use—in reflection of their own experiences—and their often-limited ability to affect change in brief street encounters. Below, we explore how members defined harm reduction, how members situated their own drug use experiences within a harm reduction framework, and the role they saw for peers within harm reduction programs.

### Defining harm reduction through lived experience

When reflecting on what harm reduction meant to them, participants described the negative impacts of drug use that they experienced and witnessed at the individual, interpersonal, and community level. Members came to this role with extensive histories of and personal experiences with drug use. For some members, this experience with drug use was their own. For many members, drug use affected all aspects of their personal lives and communities and was difficult to escape or avoid. Participant 8, who had been working as a member for two years at the time of the interview, used drugs himself and witnessed the effects of drug use on his community and family, including the overdose death of his brother.

I used to live in [omitted] from the third grade until about– let me see– from about eight years old till about in my thirties, so I done seen people use drugs. I seen people get killed. I’ve seen just about all you can see in East Baltimore. I even started hustling when I got a little older and then playing around– like I said, we started with the drinks and with the marijuana. Nineteen years old we started sniffing dope or heroin, started using heroin, and I was 38 years old before I touched cocaine. I used to sell all that stuff. Never thought I would be using any of it, but, like I say, peer pressure. I felt the peer pressure. [Participant 8, 2 years working as a member, black, male]

Other members similarly saw the impact of drug use on their friends, family, and community. Participant 4, a black woman who had worked as a member for about a year at the time of the interview, lived in a neighborhood highly affected by drug use and had multiple family members who used drugs.

My mother got high and she still on methadone… I got a sister that get high and she’s HIV-positive… So just seeing now, half of my cousins and my family members getting high that’s my– what I really know about, you know, drugs… Just seeing it everywhere I go through. By me living– most neighborhoods I lived in was impoverished… All the houses I ever had, I always lived in, it was like a major drug zone it seem like. And basically to get to your front door, you have to tell them to move off your front or go away from your door. That’s all my experiences with drugs.

Members situated this personal drug use experience within a context of structural factors, including poverty and racial segregation, that contributed to the harms of drug use in their community. Participant 1, for example, lamented that many of his friends are “either gone with the Feds, in jail, or dead” – “a whole generation now” that has been negatively affected by drug use. Participant 9 explained how criminalization of drug use had cascading negative consequences on his ability to find work.

It didn’t sit too well because of the behavior, what we had to do… to do the drugs and then the consequences of what happened once you get caught illegally. And basically in my case, that put a block to a lot of things that I was trying to do because of me developing a criminal background a record, and it wasn’t no good where, say, like if I wanted to go for a certain employment, once they did a background check and seen that I had these charges and the nature of them, I was not gonna receive that job there. [Participant 9, 4 years working as a member, black, male]

For some members, the current fentanyl overdose crisis exacerbated a long history of policy and public health neglect around efforts to address drug use in Baltimore. Participant 7, a black man who had been working as a member for two years, recalled that “dump trucks of heroin” that had flooded the city in previous decades “crippled us as a community, which exposed us [and] made us sick and dependent.” Participant 9 further elaborated that political indifference rooted in racism contributed to the contemporary overdose crisis.

Back then there wasn’t too much promotion on recovery. Or mainly the recovery thing or trying to put more assistance out there for people getting help and the struggle of addiction, you wasn’t getting too much attention at that time like you are now because it was mainly affecting the African-American areas more, the lower income more… At one time, like I said, whenever this was just affecting the blacks, it was okay, you know. But now that it’s affecting different cultures: “No, we’ve got to stop this. We can’t let this go on.”

Members used their personal experience to empathize and connect with the people they encountered during outreach. Personal experience with the broad effects of drug use formed the bedrock of members’ belief in their capacity to serve in the role of outreach worker.

My history is I’m a former addict. I used drugs. I have for my life or most of my life. And my history is that I sold drugs, I’ve used drugs, and I understand the people that I’m serving, because I’ve been there and I’ve done most of the things that they’ve done, probably worse than what they done. So I’m able to reach a large amount of people when we out doing outreach or whatever. I’m able to reach thousands of people maybe, because I was one of them, and I’m able to talk to them about trying to get them together, try to change they life around, do something different. [Participant 2, 7 months working as a member, black, male]

When asked to define harm reduction, members alternately focused solely on mitigating drug use related harms such as overdose and contextualizing it within a broader set of harms that PWUD might face. For example, one member primarily referenced the potential proximal harms of drug use when defining harm reduction.

How would I define harm reduction is– I would define it by saying it’s doing things with putting the most safety on anything you could do, trying to do it with as less harm as you can do to yourself. Being careful, being cautious about the things you do, and trying to think and do them in a smart way. Even if you’re going to get high, it’s a smart way to do it. Not sharing needles, things of that nature. Don’t be by yourself. Don’t isolate yourself. Make sure someone’s there [when you use drugs], in case something happens, like I say. And you can also test a little bit of your stuff, before you go and slam it in you, to make sure it’s not too powerful. [Participant 12, 2 years working as a member, black, male]

Members used their prior experience with the totalizing effects of drug use to explain their ability to connect with and help PWUD in multiple aspects of their lives, beyond drug use: “If you want to talk about your drug use, I can relate. If you want to talk about, again, employment, I can relate. If you want to talk about family issues, all depends, I can relate” [Participant 9].

How this experience with drug use informed peers’ own definitions of harm reduction varied. The goals and strategies of harm reduction that members advocated depended on whether they focused on proximal or distal concerns. For example, some members often specifically emphasized using drugs safely as part of harm reduction, drawing from their personal experiences as current or former PWUD.

Harm reduction to me is just talking to a person to explain to them the other options that you might have or just try to limit the risk. If they using, get some clean needles. If you using, just use bleach with your needle. If you’re using, make sure you have a Narcan kit with you. If your friend using, try to be there to support them or watch them to make sure that, if you going to use, don’t use alone. […] Not saying that you got to stop [using drugs], because that ain’t stop me from using it. I just kept doing what I wanted to do until I had to go to jail a whole bunch of time and all that kind of stuff that I actually see, because I ain’t care what nobody thought or how they saw me or none of that stuff, so I had to put myself in they shoes. But something happened with me, so if something happened with me, then it can happen with you. [Participant 2, 7 months working as a member, black, male]

From this perspective, harm reduction in practice meant empathizing with PWUD, offering them support and motivation, and giving them the knowledge and resources to use drugs safely.

At the same time members used their experience with drug use to inform how they thought about their roles as peer harm reduction specialists, many of them also had experiences with drug treatment and recovery that they drew on in their work. Other members saw harm reduction as a way to increase PWUD safety within and outside the context of drug use. One member defined harm reduction expansively to include recovery and life skills.

Harm reduction for [this organization], it incorporates a lot of things. It incorporates recovery, different kinds of recovery. It incorporates best practice, different kinds of best practice. It incorporates life in general. Professional development, you know, building leadership skills. Harm reduction with us as a whole, it’s many approaches, many approaches. So it’s not just one for the whole membership. The approaches to that, everybody approaches it different. [Participant 3, 3 years working as a member, black, male]

From this perspective, harm reduction is goal-oriented and geared toward providing PWUD with what they needed to get them on a path toward recovery.

In contrast to focusing on the proximal and immediate harms associated with drug use, other members cast harm reduction as part of a “step down” process with a gradual reduction in drug use heading toward the goal of abstinence or enrollment in a treatment program. From this perspective, continued drug use was acceptable within a harm reduction paradigm, as long as it resulted in reduction, abstinence, or treatment.

Harm reduction. Just basically meeting people at their needs. That’s all it is. Meeting them at their needs. But the main thing is to help them turn their life around from the negative to the positive. “You can do it.” Giving them that motivation and incentive. “No, you can do this. You can do this. Whatever your dream is or your goal is, you can get it done. It’s not gonna happen instantly. I’m telling you, if you’re one of those type of people that want instant gratification, no, it’s not gonna happen like that. But you’ve got to do some work, too, though.” Yeah, you’ve got to let them know that. “No, you’ve got to do some work, though,” yeah. [Participant 9, 4 years working as a member, black, male]

For Participant 9, tensions arose between the principles of harm reduction, aimed at meeting individuals at their current needs, and a desire to witness transformative change towards sobriety and abstinence. Some members perceived harm reduction as an initial step in reducing substance use and fostering safety on the journey toward abstinence and recovery. Participant 2, a Black man with seven months of membership at the time of the interview, saw his harm reduction work as a chance to “give back to the community” he had previously harmed during years of substance use, sales, and incarceration. For him, harm reduction was both a personal redemption and a means to repair community harm. This dual perspective underscores how harm reduction can serve both personal and communal healing purposes amidst broader systemic constraints. The government policies, funding requirements, and legal frameworks surrounding drug use profoundly shape organizational practices and the perspectives of peer workers, influencing what is feasible and acceptable within harm reduction and recovery contexts.

### Practicing harm reduction

Given how members’ lived experiences emphasized both the distal and proximal harms of drug use and in turn the goal of reducing drug use, members focused on helping people in moments of crisis, getting people connected to drug treatment programs, and addressing areas of need not directly linked to drug use. Members advocated for a multipronged approach to reduce harm and increase safety through both individual and community level actions that included providing information about services, working with clients to develop goals and recovery plans, or referring to additional care. They offered services that addressed a broad range of drug-related harms including overdose education and drug treatment, as well as information about housing and food assistance programs. This system of information dissemination and linkage to resources was informal, often based on the individual outreach worker’s identification of programs and services that were available. Members collected business cards and contact information from service organizations and compiled them into their own binders that they took with them during outreach events. This model afforded members a certain flexibility to recommend specific services that they may have used themselves.

For some members, the most urgent objective was to “save lives” by focusing on naloxone (Narcan) distribution and training people to use it.

Well, basically what I do, I go around and I teach people how to use Narcan. That’s basically my job. I know it’s going to be other parts eventually, but right now that’s what we’re focused on, the Narcan and people that come to us that want refills, and then we give them the refills that we have as well. [Participant 8, 2 years working as a member, black, male]

Other members prioritized providing PWUD and the community with information and resources, in addition to overdose prevention.

It’s not just the Narcan. We also can refer people to drug treatment if they want to get treatment and, you know, set people up for HIV tests; any kind of care pertaining to wellness. It’s not just– overdose prevention is one of the main things but just harm reduction in general. You know, and a lot of– part of a harm reduction is just education, people just lacking knowledge. Ignorance, you know, plays a big part in a lot. It goes on, too. Just trying to educate the community about where they can go to get help and just encourage them to get help. [Participant 6, 6 months working as a member, black, female]

For both members, the practice of harm reduction centered on addressing immediate negative consequences of drug use, including overdose and infectious disease transmission.

For other members, harm reduction required attention to distal harms often translated into an effort to get people into treatment programs, with a goal of recovery. The desire to help other PWUD reduce harms and enter recovery stemmed from members’ sense that they were role models and that their own recovery experiences created a sense of hope for the people they encountered during outreach. Members often reflected on their own past active drug use as periods of isolation from family, mental health challenges, instability, and constant worry and lives they “just don’t want to go back to” (Participant 8). Participant 11, a black man who had been working as a member for three months, recalled that when he was using drugs “nobody cared” about what would happen to him, and it was only after he was incarcerated that he saw the possibility of recovery.

Well, when I see how– that people are literally just tearing themself down, physically, mentally, morally, spirituality. I mean, it’s really bothersome to me and every time I see it, I– for some reason, I reflect back to myself. Because I say wow, I was really like that. You know, I was– and then, in a lot of cases, I have to even say to myself you was even worse than some of that… I start seeing some positive things, some hope, some people that really– their own way of saying that they’re sick and tired of being sick and tired. And I start seeing that and I remember, yeah, I used to be like that and I could be like that again if I allow myself. And then, that makes me want to reach out even more. [Participant 11, 3 months working as a member, black, male]

For this member and others like him, harm reduction was not only about helping people avoid the immediate, proximal negative consequences of drug use. Rather, they expressed a need to help the people they encountered during outreach stop using drugs and undergo a personal transformation.

Members used the “readiness” model to reconcile potential contradictions between the harm reduction principle to nonjudgmentally “meet people where they are” and their own desire to see clients enter treatment, as they had done. One member explained his role in helping PWUD as “opening the door,” but concluded that it was up to the individual to decide what to do with the information or services being offered. Members invoked the metaphor of a journey – with a destination of recovery or abstinence – to explain how they helped the people they encountered.

I guess the one challenge is– well, it’s not really much of a challenge anymore, but it still is a challenge, because you have many pathways to recovery now, and so earlier on a lot of these pathways were not accepting helping people while they’re still using. And sometimes it can still be in certain arenas and certain pathways like, “Well, you can’t do this while you come here” or “You can’t express this” or, you know, so I guess that kind of maybe can be considered a challenge. [Participant 7, 3 years working as a member, black, male]

In practice, this strategy meant that some members did not initiate conversations about harm reduction or abstinence but shared their experiences and resources when prompted.

Eventually they’re going to get tired of what they’re doing and they’re going to come ask me “Well, how did you get clean,” and then I can tell them. But until they come to me I can’t say anything. All I can do is live the life, but that is the best way to do it. That’s the best way to get somebody to come off drugs. Don’t even mention it: “Hey, have fun but you be safe.” Eventually you’re going to get tired of shooting that garbage and then you’re going to come ask me, “How did you get off of drugs? How did you do this or how did you do that?” [Participant 8, 2 years working as a member, black, male].

Members described harm reduction as “meeting people where they are at,” but ultimately, members hoped the end point of that path would be abstinence, drug treatment, or reduced drug use for the PWUD they engage.

However, being open to engage with people who are actively using drugs could be challenging for members in recovery and create a new form of harm for the members themselves. Participant 9, who had been a member for four years, described that seeing people use drugs “can look pretty good, pretty attractive” and forced him to actively work against desires to use and limited his interactions with people he encountered during outreach.

That would make some thoughts come up. No, no, no, no, no, no. But, like I said, I don’t talk down to them. I’m not gonna do that. I just remove myself away. I may have to hold a quick conversation with you at that time, but I would get that– I would just remove myself. But if I see there’s something physical going on with you or something mentally ain’t right, I’m gonna stay there to help you to get through it at that time. I’m gonna stay there to help you get through it, if I can, or I’m gonna try to get you some help.

For this member, encounters with people actively using drugs posed a potential threat to his own recovery and motivated him to limit how much he engages with people during outreach. At the same time, he recognized that people needed help and was willing to help in any way that he could while protecting his sobriety. In contrast, Participant 6, who had worked as a member for 6 months, said that seeing people use drugs motivated her to maintain her sobriety.

Personally, when I see people who are using, I’m so grateful that it’s not me. They keep me grounded. They keep me going to my meetings, doing whatever I need to do to just stay on track. And actually, they help me stay on track more than anybody, because I don’t ever, ever want to live that, you know? Never. So that’s good for me. It works good. That’s like a good deterrent for me.

The baseline objective of members’ outreach was distributing Narcan to prevent overdose, which was relatively standardized among members. But members also offered a broad range of other services and referrals beyond Narcan or other drug resources. The provision of and referral to these other resources was informal and largely based on personal experience, knowledge, and comfort level (i.e., talking to high PWUD being hard for some people trying to maintain their own abstinence), which allowed for the flexibility to adapt to an individual’s circumstances, but also contributed to inconsistencies between how different members practiced harm reduction in the field. Some offered drug treatment, others waited for it to be brought up to them. Some had a range of social services that they were familiar with and referred people to, while others had a more limited set of resources they could access.

## Discussion

Many of the participants in this study had personal, direct experience with substance use, typically their own but also that of their close friends and family. They were also deeply steeped in the principles and practice of harm reduction through formal training and certifications, networking with other harm reduction practitioners at the local and national level, and in their own outreach. They drew on this experience with dependent drug use, recovery, and harm reduction in their interpretation and practice of harm reduction and how they understood their roles as outreach workers and harm reduction specialists. Members embraced an expansive definition of both the harms associated with drug use and possible harm reduction strategies beyond, provision of safe use supplies such as naloxone and test strips. They lived in the tensions between their role as helping others minimize the proximal harms of drug use and their ideas and experiences of harm and recovery. They respected other people’s drug use but were actively living in a place between drug use and sobriety. Many of the members were either in recovery themselves or, if they were actively using while providing harm reduction services, had a goal of reducing or quitting their own drug use. They did not see these two concepts—harm reduction and recovery—as incompatible. Rather, they understood drug use as something dynamic with periods of more and less use, interspersed with periods of abstinence, and talked about harm reduction to keep people safe until they could begin the process of recovery.

While the formal definition of harm reduction specifically indicates that eliminating drug use or ensuring abstinence is not a goal, harm reduction specialists in this study almost universally placed harm reduction within a recovery framework. They understood that the path to recovery was complicated, nonlinear, and rife with barriers or relapses. Informed by their own experiences of feeling “outside” of society during periods of their most active drug use, members framed harm reduction from a recovery standpoint, suggesting that it could be used to help people undergo a process of decreased drug use or abstinence and therefore become active participants in society [22, 23]. At the same time, members did not embrace an overly simplistic idea of recovery that assumes people who use drugs have control over their drug use. As their efforts to connect people with a range of resources showed, they recognized the role of social determinants of health on drug use-associated proximal and distal harms. By recognizing the heavily influential racial and social factors underlying drug use that produce harms, members were able to provide continued support for those actively using drugs with a nonjudgemental and non-stigmatizing mindset as well as a deeper understanding of people’s environments. It is from this position that members were waiting for the people they encountered during outreach to indicate their readiness for recovery.

Often using the language of “readiness” to bridge two seemingly incompatible approaches to drug use—harm reduction and recovery—peers simultaneously tried to address the structural factors in their roles as information providers and resource connectors. Peers ideas about harm reduction and recovery countered overly simplistic models of behavior change and notions of individual willpower that underpin some approaches to drug use recovery [14, 23]. When members explained what they understood harm reduction to be, they typically spoke about the need to address the “whole person,” but the nature of what this meant and how this conception was enacted varied. They often took an approach to harm reduction that was not dogmatic. For some, this vision of harm reduction meant that any harms that a PWUD might encounter, including but not limited to those associated with drug use, could be potential targets of intervention. For others, they focused specifically on drug use-related harms, but they contextualized these harms within a broader context that included factors that contributed to drug use, family circumstances, and even the economic conditions of the neighborhood in which drug use occurred. There was a universal recognition among study participants that more needs to be done to address the broad needs of PWUD.

Some of the members expressed ideas about how to help PWUD that prioritized their own experiences and ideas about what constitutes a meaningful, productive life, rather than centering clients’ needs. They held themselves up as examples, particularly their work as peer harm reduction specialists, of how the outcome of eliminating drug use could be achieved. This mindset is a potential source of conflict with foundational principles of harm reduction. This incompatibility originates from policy frameworks that prioritize drug treatment and recovery over harm reduction [1]. These policies often allocate funding and resources predominantly to programs that promote abstinence-based treatment models, reflecting societal norms and political priorities that favor moralistic approaches to drug use [10, 20, 36]. Consequently, harm reduction initiatives, which aim to reduce the immediate risks associated with drug use rather than eliminate it entirely, receive less support and face greater scrutiny. This imbalance perpetuates a dichotomy where harm reduction strategies may be marginalized or underfunded despite their proven effectiveness in reducing HIV transmission, overdose deaths, and other public health risks associated with drug use. At the same time, the incorporation of recovery narratives into harm reduction approaches is not uncommon. For example, Lee et al [24] found that staff at two harm reduction programs viewed harm reduction and recovery-based approaches as complementary. Thus, the conflict between harm reduction and recovery-oriented approaches is both philosophical and deeply embedded in broader social and policy contexts that influence resource allocation and public discourse on drug policy.

Members’ interpretation and enactment of harm reduction parallels the recommendation proposed by Ashford et al. [2], who argue that from a policy and programming perspective, hybrid programs and service models that incorporate both harm reduction and recovery might best meet the needs of PWUD. They may also facilitate relationships in which harm reduction can be an effective entry point for people interested in receiving treatment. Ashford et al. [2] noted that peer-based recovery community organizations (RCOs), largely supported by federal grants, provide recovery-focused support services such as recovery coaching. RCOs can also serve as information and referral clearinghouses for other substance use treatment programs and social support services, such as housing [2]. Harm reduction services such as needle exchange, however, are typically *not* part of RCOs given the RCO focus on initiating recovery and eventual abstinence. A recommendation to create hybrid programs reflects the experiences of the peer harm reduction specialists in our study. Harm reduction programs can provide an important point of contact between PWUD and service providers. However, harm reduction is often taken as a principled stance in opposition to abstinence and recovery. How people with competing models or interpretations of harm reduction, often based on lived experience, can be incorporated into harm reduction-based programs needs to be addressed. The members in this study had complex interpretations of harm reduction that may not align with traditional definitions of this approach, raising questions about how peers’ own dependent drug use and recovery experiences can be embraced.

While working as a peer and embracing one’s own journey can be rewarding or helpful to one’s own recovery identity, members also faced significant challenges in their work. Peers involved in harm reduction programs may be actively using drugs and may have exposure to traumatic events such as witnessing or experiencing fatal or non-fatal overdoses. Peers working in recovery settings may risk their own sobriety through continued engagement with people actively using drugs and as a coping mechanism for stressors related to the work itself. Peers may experience compassion fatigue, overextending themselves beyond employment parameters, and incessant emotional demands [48, 52]. Within the workplace, they may experience power imbalances, stigma against former or current users, unclear role distinction, and perceived lack of professional development and advancement, all of which can lead to burnout and trauma [16]. The organizations that employ peers may not recognize the stressors peers encounter doing harm reduction work and recovery coaching, and there may be little support to address these stressors [32].

Organizations across the substance use service landscape must address the unique needs and questions that working with peers engenders. Organizations also differ on the amount of training they provide peers, how they are integrated into the organization, compensation structures, and support. In addition, minority communities in urban areas have been historically underserved by harm reduction and high-quality treatment programs, and peer-based approaches have the potential to fill this gap [35]. These differences express an organization’s values (e.g., prioritizing sobriety and professionalization over more proximal experience with the target population) and raise questions about whether the appropriate expertise and knowledge is being harnessed and how best to help peers succeed in their roles. How current and former PWUD from marginalized communities can be successfully integrated into service-provision roles is not clear.

This study had several limitations. First, the experiences and perspectives of the members reflect a single peer-based program that primarily conducts street-based outreach. It does not include the experiences of peers working in other settings, such as fixed site syringe services programs or treatment centers. Peers in these settings likely have different understandings of drug use, harms, and recovery and how they should relate to their clients. Second, data were collected just as the drug market transitioned from heroin and prescription opioids to one dominated by fentanyl and xylazine. Given the very different harms associated with these drugs, harm reduction priorities and strategies have likely evolved. Finally, this analysis did not explore dynamics around diverse interpretation and practice of harm reduction within the organization itself, for example between frontline staff, supervisory staff, and managers. Such an analysis could provide valuable insight into how organizations reconcile value conflicts, create cohesion, motivate and engage employees, and make decisions about service provision and growth.

## Conclusion

In the context of the current opioid epidemic, peers continue to be critical frontline workers. Members expressed tensions between their own sobriety, challenges with drug use, belief in harm reduction, and continued drug use of fellow members and the people they encountered during outreach. This complex understanding of the relationship between harm reduction and recovery points to a need for peer harm reduction training and program models that present alternative models for understanding dependent drug use and treatment and empowers peers to address structural factors via a broad range of accessible and referrable social services, including housing support, comprehensive health care, and drug treatment, if desired. The tensions peers face in their work underscore the importance of training peers around the various dimensions of harm reduction, empowering peers to holistically help clients in multiple ways, and supporting peers’ emotional and physical well-being. Finally, these tensions underscore the need for systemic policy and funding reform to decrease divisions between harm reduction and treatment.

## Data Availability

The data generated and analyzed during this study are not publicly available due to concerns about data privacy and participant confidentiality associated with a small study sample in a highly specialized and localized environment.
